# Understanding Italian Consumers’ Perception of Safety in Animal Food Products

**DOI:** 10.3390/foods11223739

**Published:** 2022-11-21

**Authors:** Maria Piochi, Michele Filippo Fontefrancesco, Luisa Torri

**Affiliations:** University of Gastronomic Sciences, 12042 Pollenzo, Italy

**Keywords:** citizens’ attitude, behavior, food technology neophobia, risk, milk, honey, eggs

## Abstract

The concept of food safety is still underexplored among consumers, especially in relationship with the perception of food technology. Through an online survey (*n* = 489), this study explored: I, how perceived safety is related to products obtained with different technological treatments and described with different commercial information; II, the role of food technology neophobia (FTN) in consumers’ safety perception of animal food products. The technological transformation and commercial information significantly affected the perceived safety in all product categories. Milk and eggs were associated with a high number of perceived hazards (with similar patterns), while honey to the lowest. The certification ‘organic’ positively affected the safety perception of eggs and honey. With the increase of the distance in product origin (local/regional vs. Extra-European) the perceived safety consistently decreased. FTN affected the perceived safety of milk and eggs, depending on the degree of familiarity with the technologies of production. Highly FT neophobic people are perceived as less safe than low FT neophobic people with few familiar products with a higher technological degree of transformation. Results expand the knowledge in people’s attitude towards animal products, particularly considering the technology perception. The outputs may interest policy-makers and food companies, in rethinking the communication strategy concerning food safety.

## 1. Introduction

The concept of ‘Food Safety’ refers to all those hazards, whether chronic or acute, that may make food injurious to the consumers’ health and it is related to microbiological hazards, pesticide residues, misuse of food additives, chemical contaminants, including biological toxins, and adulteration [1]. Food safety is a vital issue for public health and a daily concern for consumers, producers, and any public/private stakeholders. In fact, contaminated food can harm people, increasing demand for health services, insurance, and government expenditures on public health and other social costs and can transmit diseases and even kill [2]. From a citizens’ perspective, food safety is an element on which they cannot compromise (in other terms a pre-requirement) [3]. The perception of safety is linked to consumers’ food safety risk perception defined as the individual’s perception of the presence of an attribute (safety) in food and the probability and severity of health consequences of its consumption [4]. According to a recent report by the European Commission [5], Europeans have a high level of awareness of food safety topics, with at least 60% of respondents being familiar with pesticide residues in food, diseases found in animals, allergic reactions to food or drinks, similar or steroid residues in meat, food poisoning from bacteria, and environmental pollutants. However, the impact on European citizens’ perceptions and attitudes toward safety and food integrity (not adulterated) has not been systematically evaluated [6]. Perceived safety has been mostly studied in relationship to single products such as milk [7], meat [8], or honey [9]. However, studies exploring the perception of safety in the same participant group, thus allowing a comparison across products, are lacking.

The perception of safety can influence human behaviors (such as willingness to pay, choice, etc.) by inhibiting or pushing toward the consumption of a specific food. Thus, understanding consumers’ perception of food safety is relevant to help in designing guidelines to improve both communication activities and citizen behavior related to food safety. The reasons beyond consumers’ behaviors with respect to food safety and risk information were reviewed [3]. Currently, there is an increasing possibility for citizens to be exposed to information related to food safety and new risks, and the COVID-19 pandemic sharpen the importance of information sources [10]. On the other side, novel technologies are increasingly entering the food sector [11,12], ending up in some cases in products obtained through specific technologies that identify precise commercial classes. Some of these products are already well established among European consumers, such as (Ultra High-Temperature) UHT-milk [13], while others are still less spread (such as some non-thermal technologies such as cold plasma, ultrasound, high-pressure processing, irradiation, pulsed light, etc.; see [11] for a review). In general, despite the possibility to improve the nutritional quality of products obtained through recent technologies and although offering in many cases reduction of energy demand, thus being environmentally sustainable [14], consumers may be cautious of food products produced using these new techniques [15]. This is rooted in a difficult cultural negotiation that indicates technology as an ambivalent resource that can enhance food granting stronger security and flavors, but at the same time distances the product from an ideally positive status of naturalness [16]. The Food Technology Neophobia scale (FTNS) was developed in 2008 and identifies neophobia in relation to food technology [17]. FTNS includes four dimensions (perceived risk; uselessness of technology component; benefits and health effects; trust in media role). Factors determining neophobia and neophilia with regard to new technologies have been recently reviewed [18], showing that the FTNS is a better instrument for predicting individuals’ willingness to approach foods produced using novel technologies than other available scales [18]. However, studies on the effect of food technology neophobia in specific product categories envisaging different products characterized by different technological treatments are limited. 

Thus, the current paper aimed to: (i) Compare the perceived safety of three different product categories with animal origin (milk, eggs, honey) and different frequencies of consumption in the same group of consumers; (ii) Explore the relationship between the safety perception and different technological treatments and commercial information; (iii) Investigate the role of food technology neophobia on consumers’ safety perception. The study focused specifically on animal (not vegetable) products because it is known that animal-originated foods are perceived as riskier than vegetal ones [19]. Moreover, in animal products, the relationship between an individual’s perception of safety and the probability and severity of health consequences of its consumption is more correlated with willingness to buy (thus it is more strongly related to a behavior), than in vegetable products [2]. The three food matrices were chosen for different reasons. In particular, they can similarly either be purchased as such or can be used in food transformation (e.g., especially in the bakery sector). They have a crucial economic value in North Italy [20], being characteristic of proximity productive chains (characterized by a high closeness among the stakeholders involved—also identified as ‘km 0′ productive chains), and they are recently under-discussed in the news for being object of food frauds that can undermine the perception of safety in the public opinion (the total economic value seizure (€) for national frauds in 2021 was higher than 14,000€ according to the Italian Department of central Inspectorate for Fraud Repression and Quality Protection of the agro-food products and foodstuffs [21]).

## 2. Materials and Methods

### 2.1. Online Survey

An online survey was developed in the Italian language using the QuatricsXM^®^ platform (Provo, UT, USA) and sent on a national level by email to the staff, students, and contacts of the University of Gastronomic Sciences (Pollenzo, Italy). The link was also spread through social media (Linkedin, Instagram, Facebook, and Twitter) and word of mouth. The survey included questions related to respondents’ sociodemographic characteristics: age, sex, living context (Country/Rural context < 10.000 inhabitants; Medium-sized cities, 10.000–70.000 inhabitants; City > 70.000 inhabitants), nationality, Italian region, diet (Omnivore, Flexitarian, Vegetarian, Vegan) and frequency of consumption of the three products (daily, at least once a week, at least once a month, less than once a month). Perceived safety was asked in each product category (milk, eggs, honey) for different items, each corresponding to a specific technological treatment and/or a specific commercial type (Table 1). 

Then, the perceived safety of products with different origins (local/regional, Italian, from the European Union (EU), from countries outside the EU) was rated on a 9-point scale (1 = not at all safe, 9 = extremely safe). For each product, the most dangerous hazards for human health were explored with Check-All-That-Apply (CATA test). CATA test is a consumer-friendly method in which the participant is provided with a list of attributes and he/she must select all the attributes that are appropriate to describe the product [22] (*Select the hazard(s) for human health that are most dangerous in the product (more options possible): Environmental contaminants (heavy metals, mycotoxins, herbicides, etc.); Antibiotics; Microorganisms (Bacteria, viruses, molds, yeasts, …); Incorrect storage conditions after purchase; the product does not present any source of risk; I don’t know*). The three sections of questions related to the three products (milk, eggs, honey) were randomized across subjects. Finally, the Food Technology Neophobia scale was administered [17]. The survey took 15–20 min to be completed.

### 2.2. Respondents

A total of 681 people accessed the survey. Including only subjects who completed the questionnaire, 489 respondents were finally considered (females = 66%, age range = 18–84 years old, mean = 41 years old) (Table 2). The whole experimental procedure was approved by the Ethical Committee of the University of Gastronomic Sciences (Ethics Committee Proceedings n° March 2021). The work was carried out in accordance with the international ethical guidelines for research involving humans established in the Declaration of Helsinki. All subjects gave their informed consent before answering the questionnaire.

### 2.3. Data Analysis

Descriptive statistics were applied to all variables. Values are expressed as the mean and standard error of the mean in the text. Subjects were grouped into three age classes respectively: 18–30 years old, 31–50 years old, > 50 years old. The effect of sex was assessed on the perception of the safety of product category (considering milk, eggs, and honey as levels) with a two-way Analysis of Variance (ANOVA) model (fixed factors: product category, sex; model with interactions). Identical models were used to assess respectively the effect of age class, living context, and diet on perceived safety. To assess the impact of the technological transformation and of the main commercial information given on labels on the perceived safety ratings within each product category, three ANOVA models were separately conducted in each product category (fixed factor: level of technological transformation or commercial type; random factor: subject). The effect of the origin (local/regional; Italian; European, outside the EU) on the perception of safety was separately assessed for each product category with two-way ANOVA models (random factor: subjects; fixed factor: origin; model without interactions). For each product category (milk, eggs, honey), the total number of the chosen hazards was computed across all subjects from the CATA question ‘*Select the hazard(s) for human health that is most dangerous in the product (more options possible*’ (total number of chosen hazards). The higher the number of hazards chosen the higher perception of risk. A Chi-squared test was applied to assess whether the percentage of subjects choosing the main hazards within each product category significantly varied, combined with a Cramer’s V test to assess the effect size. To analyze results from the Food Technology Neophobia scale (FTNS), a final score was computed for each subject by summing ratings given to items (after having reversed appropriate items [23]). Based on the quartile values of FTNS, subjects were classified into three classes (low, medium, and high). The effect of food technology neophobia was assessed on the perceived safety of each item for all product categories. Three one-way ANOVA models were separately conducted to assess the effect of FTN on the perceived safety of each product category (fixed factor: level of technological transformation or commercial type). Each ANOVA model was followed by a Tukey’s HSD test (*p* ≤ 0.05) to check for significant differences between mean values and by the computations of effect sizes with eta^2^ for each factor constituting the ANOVA models (η^2^ = Sum of square of the effect/Sum of squares total) and partial eta^2^ for the whole model (partial η^2^ = Sum of square of the model/(Sum of square of the model + sum of square of error)). The complete ANOVA models, including effect sizes, used in the study are reported in Supplementary Materials (Table S1). Correlations across variables were studied with Pearson’s coefficients. All analyses were conducted with XLSTAT version 3.1 software (Addinsoft, New York, NY, USA).

## 3. Results

### 3.1. Sample Composition and Generalities on Safety Perception

The population of respondents had a prevalence of females it was balanced in terms of both age classes and context, and it was mainly composed of omnivores (Table 2). For sex, 2 subjects answered ‘I prefer not to declare’ and 4 subjects declared ‘Other’ (not included in Table 2). As expected, in the population the familiarity with the three products (milk, eggs, honey) varied greatly: the most frequent response was respectively ‘Daily’ for milk, ‘Minimum 1 per week’ for eggs, and ‘Less than 1 per month’ for honey. The perception of safety strongly varied based on the product category considered (F = 57.01, *p* < 0.001): eggs were considered less safe (6.2 ± 0.0) than the other two product categories (milk: 6.6 ± 0.0, honey 6.7 ± 0.0). Sex significantly affected the perceived safety (F = 61.60, *p* < 0.001) of the three products with a medium-large effect size (η^2^ = 0.4); males gave significantly higher average ratings of perceived safety, than females, thus they were more deemed to consider the three products safer. However, no significant interaction sex*product was found in terms of perceived safety. A negative and weak significant correlation was found between age and perceived safety of the three product categories considered together (r = −0.030, *p* = 0.003). Age classes significantly affected the perceived safety (F = 40.40, *p* < 0.001) of the three products, with significantly different average values across the three age groups (31–50 years old > 18–30 years old > over 50 years old) and a medium-large effect size (η^2^ = 0.4). However, no significant interaction between age class*product was found. Interestingly, the interaction living context*product category was also significant and affected the perception of safety (F = 2.54, *p* < 0.05): eggs were perceived as significantly less safe in all contexts, but in rural contexts, the honey was perceived as the safest product, while in cities milk tended to be the product category perceived as safest. Classes for diet were not balanced in number (vegans and vegetarians were muchly underrepresented), which was however close and similar to the Italian condition, with approximately 8.2% of vegetarians or vegans in 2021 according to the Research Institute of Italians (Eurispes) [24]. The diet significantly affected the perception of safety (F = 33.75, *p* < 0.0001) with a large effect size (η^2^ = 0.5): omnivores and flexitarians gave significantly higher ratings (6.5 ± 0.0 and 6.5 ± 0.1, respectively) than vegetarians (5.8 ± 0.1) and vegans (5.0 ± 0.3). Moreover, a significant interaction type of diet*product category was found (F = 12.30, *p* < 0.0001): differently from omnivores and flexitarians both vegetarians and vegans perceived eggs significantly less safe than other groups and less safe than other product categories (milk, honey).

### 3.2. Effect of Technological Transformation on Safety Perception and Perceived Hazards

**Figure 1:**
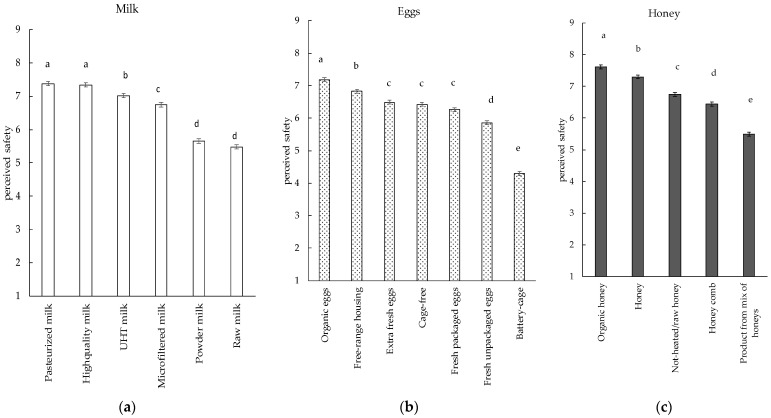
Perceived safety of different commercial types of milk (**a**), eggs (**b**), and honey (**c**) from 489 respondents. In each figure, different letters indicate statistical different mean values from two-way ANOVA models (fixed factor: level of technological transformation or commercial type; random factor: subject).

The product category (technological transformation and commercial information) significantly affected all product categories: milk (F = 156.81, *p* < 0.001), eggs (F = 246.27, *p* < 0.001), and honey (F = 186.07, *p* < 0.001), with small effect sizes (η^2^) in milk and honey (0.1) and in eggs (0.2). In the population of respondents, pasteurized and high-quality milk were perceived as the safest, while powdered milk and raw milk as the least safe (Figure 1a). UHT milk was perceived as significantly safer than microfiltered but less than pasteurized and high-quality milk. For both eggs and honey, the information related to the organic certification was perceived as the significantly safest. For eggs, the type of hens breeding was clearly associated with safety (Figure 1b), while hens from outdoor breeding were perceived safer than hens from free-range breeding. ‘Battery cage’ eggs were perceived as the least safe. For honey, the product highly handled (product from a mix of honeys—which requires at least a blending of different batches) was perceived as the least safe (Figure 1c).

**Figure 2:**
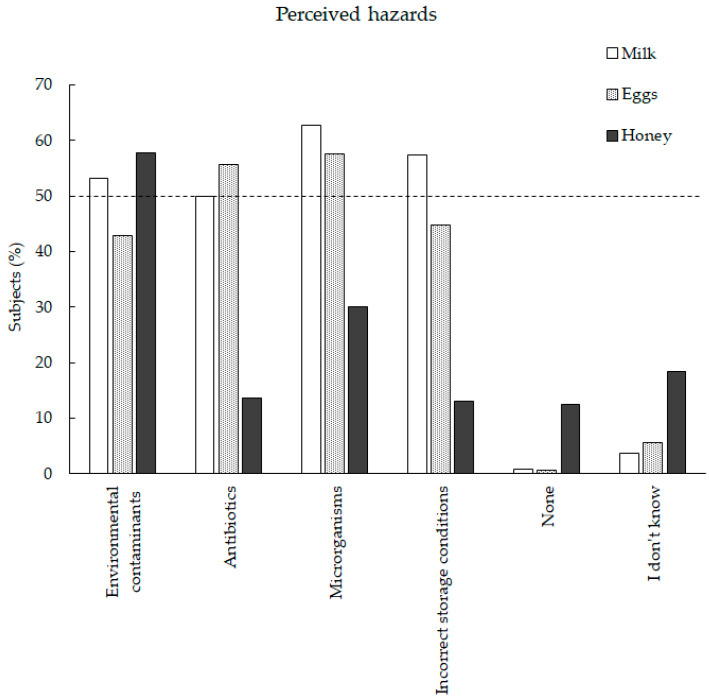
Percentage of respondents (*n* = 489) selecting characterizing hazards for the three considered product categories. Note: the dotted line indicates hazards chosen by at least 50% of respondents.

The perception of the main hazards in the population for the three product categories is summarized in Figure 2. The chi-squared test highlighted a strong difference across products of the main hazards (χ^2^ test 18.31, DF 10 = 97.05, *p* < 0.001), with a medium effect size (Cramer’s V test = 0.3). The total number of chosen hazards was respectively: 712 for honey (being the lowest), 1124 for milk, and 1013 for eggs. Differently from honey, milk and eggs had similar patterns of chosen hazards, since both had a high similar number of choices for: ’Antibiotics’, ‘Microorganisms’, and ‘Wrong conservation’. In milk, the main hazards (chosen at least by 50% of respondents) were: ‘Microorganisms’, ‘Wrong conservation’, ‘Environmental contaminants’ and ‘Antibiotics’. For eggs, the main chosen hazards were ‘Microorganisms’ and ‘Antibiotics’ whereas for honey the main hazard was ‘Environmental contaminants’. Moreover, honey was perceived by participants as not affected by wrong conservation nor by the presence of contaminants (below 20% of respondents). Lastly, honey was the product with the highest percentage of respondents associating it with any contaminants (’None’) and uncertainty (‘I do not know’).

Independently from the product category, the origin (local/regional; Italian; European, Extra-European) had a strong significant effect (*p* < 0.0001) on declared safety. In all product categories, the same significant trend was observed (milk: F = 724.21; eggs: F = 763.11; honey: F = 695.11; with medium effect sizes in all cases, η^2^ = 0.4): the local/regional product was perceived as significantly the safest, followed by the Italian, then the European and finally the Extra-European. Thus, the increase in the distance between the origin and the consumption site negatively affected the perceived safety.

### 3.3. Effect of Food Technology Neophobia on Perceived Safety

The sum of ratings for FTNS ranged from 23 to 85, with a median of 52. According to quartile values, respondents were classified into ‘low’ (FTNS ≤ 45), ‘medium’ (FTNS 46–60), and ‘high’ (FTNS ≥ 60) food technology neophobic subjects. FTNS was slightly and negatively correlated with age (r = 0.11, *p* = 0.01). As expected, with the increase of FTNS the perception of the safety of some items belonging to certain product classes decreased (Table 3), suggesting a higher perceived concern for the safety of the interesting products. The significant effect of FTN status was observed for milk (apart from the item ‘High-quality milk’) and for eggs (for the items: ‘Fresh packaged eggs’, ‘Extra fresh eggs’, ‘Free-range housing’), but not in honey. In particular, high food technology (FT) neophobic subjects perceived raw milk as safer than the other groups. For a more familiar product such as ‘Pasteurized milk’ no differences were found across groups. As expected, for the three highly-technologically transformed type of milks (‘UHT milk’, ‘Powder milk’; ‘Microfiltered milk’), low FT neophobic participants rated the perceived safety as significantly higher than high FT neophobic people. Also, for eggs, the same trend was observed for all significant items: low FT neophobic participants rated the perceived safety as significantly higher than high FT neophobic people. For honey, the three groups did not significantly differ in mean ratings for any item, and mean ratings were modest (‘Products from a mix of honeys’, ‘Not-heated/raw honey’, ‘Honey comb’) or high (‘Honey’, ‘Organic honey’) in all groups. For all the items (commercial types) for which a significant effect of FTN status was found, the effect sizes were low (equal to or lower than 0.1).

## 4. Discussion

### 4.1. Perception of Safety in the Population in Respect of Socio-Demographic Factors and Diet

Considering the perception of safety for the three product categories (milk, eggs, honey), we found a negative correlation between age and perceived safety and that females gave significantly lower ratings than males. Both these results were expected and in agreement with a recent study showing that risk perception (considering a list of generical items valid for different food) increased with age and women perceived greater food safety risk [25]. The effect of gender is well documented. For example, in females, the food safety label moderated the effect of risk perception on perceived quality, while in males this effect was not significant [26]. Regarding the living context, rural and urban contexts may bring some differences in lifestyles and food behaviors [27], that in the specific case of Italian territory may translate into a different familiarity towards some product/process of production (for example honey) which may have been perceived it as safer, since it is known that the familiarity has an effect on the risk and benefit perception [28]. Despite the complexity of reasons that push people towards a vegetarian [29] or vegan diet [30], people adherent to both diets have generally a higher rejection of animal-based products. This can negatively influence also the perception of safety. Differences between omnivores and vegetarians were found in personality traits, values, and empathy; with vegetarianism, it is associated with greater openness and empathy [31]. To the best of our knowledge, no direct comparison in the safety perception between omnivores, vegetarians and vegans was conducted. However, based on the above-mentioned literature on the perception of vegans and vegetarians for animal-based products, the more negative judgment of the perceived safety of animal-originated products found in the current study was expected. As previously specified, even if the number of vegetarians and vegans was low in our study, the fact that it was comparable to the percentage of these people in Italy and considering the high effect size eta^2^ found from the ANOVA model on the impact of diet, made us being confident that these results are reliable.

### 4.2. Impact of Technological Transformation on Perceived Safety

In the product category of milk, the two types of pasteurized milk (‘Pasteurized milk’, ‘High-quality milk’) were perceived as the safest, followed by UHT milk. In Italy, within the category of dairy products, UHT milk had the 13% of the overall market share and fresh pasteurized milk 6% [32]. So, both products (pasteurized and UHT) are common and familiar in the population. Instead, the two least safe items were ‘Raw milk’ and ‘Powder milk’, which represent two extremes in terms of technological transformation (meant as the number of unit operations applied to the food matrix to achieve the final wanted product). In particular, raw milk does not undergo any thermal treatments higher than 40 °C [33]. In Italy, raw milk can be sold in any region, through distributing machines that must indicate the indication “Product to be consumed only after boiling” according to according to the ministerial decree (2009) [34]. In the current paper ‘raw milk’ was associated with a higher greater food safety risk probably due to the fact that the consumption of raw milk according to literature is still controversial due to possible contaminations with pathogens that may originate foodborne diseases (*L. monocytogenes*, *Salmonellae spp, E. coli*, etc.), despite the numerous biodiversity and nutritional properties [35]. From a technological perspective, powder milks are derivatives of milk obtained from several unit operations (i.e., pasteurization, the concentration of total solids by evaporation, and spray drying) [14,36]. As expected, the powder milk, being the product mostly transformed from a technological point of view, was perceived as the least safe. Indeed, among the three considered product categories, milk was the only one in which the technological transformation (mainly including the thermal treatment) clearly play a clear role, by giving rise to different commercial classes depending on the treatment they undergo. Therefore, milk was the product that mostly allowed to highlight the effect of the technological transformation on perception of safety. 

Eggs are meant as eggs in the shell—other than broken, incubated, or cooked eggs—that are produced by farmed birds and are fit for direct human consumption or for the preparation of egg products [33]. Generally speaking, eggs are classified as the riskiest group of products in several European countries [19]. Eggs are a product category not characterized by any technological treatment. However, we found a clear diversification in perceived safety, based on information related to aspects of production (animal welfare, certifications, etc.) that are commercially used. For example, the system of animal breeding (‘Battery cages’) was clearly associated with safety aspects from a consumers’ perspective with negative meaning, while the housing systems which are more respectful of animal welfare (such as the ‘Organic’, and ‘Free-range housing’) performed clearly better than the ‘Battery cage’. Eggs and laying hen housing have received significant attention in recent years. Indeed, it was shown that the hens’ system of housing differently capture the attention of consumers online (number of posts on social media) and the cage-free and free-range had the greatest number of online hits in the searches conducted [37]. For eggs, the mentions ‘Cage-free eggs’, together with ‘no antibiotics given to hens’, are among the newest trends [38]. 

In honey, the perceived safety has been poorly explored. A few reports showed that honey, being one of the traditional and complementary healthcare used for upper respiratory tract symptoms, especially in children, is believed to be natural and safe but in some cases, some dangers (such as from *C. botulinum* contamination) may be underestimated [39]. A previous study with Italian participants showed that the ‘organic’ attribute was more important than other factors, such as the place where the honey was produced (landscape), but less important than the country of origin [40]. Congruently, in our case, the attribute ‘organic’ had a very positive meaning for the perceived safety of honey. For honey, as found for milk, the perception of safety was negatively influenced by some production treatments (mixing—‘Product from a mix of honeys’) while the lack of technological transformation (the non-heating—‘Non heated/raw honey’) was seen as positive.

In general, for all three product categories, items were perceived as safest if having a local/regional origin. These findings were expected, since the local dimension has become increasingly important in food products [41]. Moreover, the fact that consumers positively perceive products from short productive chains may push producers to reduce distances in the supply chain.

We consider now the main hazards characterizing each product category. Indeed, the knowledge about food safety topics and main hazards greatly varies across European countries [5]. The current study considered a sample of Italians, and the population seemed quite aware about the main hazard in milks. Similarly, a report showed that despite 66% of consumers being misinformed about regulations, most (90%) were conscious of the risks of raw milk consumption [7]. In the current study, ‘Microorganisms’ were the first perceived hazard in eggs. That shows a good awareness among consumers, since egg spoilage is a microecological phenomenon, involving several specific spoilage microorganisms [42]. Considering honey, it presents some hazardous components that are both well known (for reviews see [43]) and less known, such as plant toxins [44]. People often believe that ‘natural food’ is harmless and honey, as a natural nutritious sweetener, falls into this category, while certain honeys may be toxic due to their high content of toxins [44]. In the current study, ‘Environmental contaminants’ was the primary hazard source perceived, and this again showed a good awareness among citizens, since pesticides [45] and heavy metals (e.g., lead, arsenic, and mercury [46]) are hazardous components in honey and can be considered environment contaminants. 

### 4.3. Effect of Food Technology Neophobia

The effect of FTN was clear, especially on the safety perception of the product with a clear commercial classification reminding of technological meaning (milk). In this case, FTN clearly played a role in the expected direction only for items either with a clear technological valence and a low familiarity (example: ‘Microfiltered’ or ‘Powder milk’ or ‘UHT milk’). While for items having a technological valence but highly familiar (such as ‘Pasteurized milk’) there was no difference in perceived safety between groups with different FTN. That seems in agreement with a previous study suggesting that people are programmed from early childhood to prefer familiar foods and FTN is one of the main barriers in accepting novel and unfamiliar foods [47]. The fact that highly food technology neophobic respondents perceived raw milk as safer than the other groups was interesting. Since from safety aspects raw milk is considered riskier than sanitized milk [35], either highly technology neophobic people have a lower knowledge about the meaning of thermal treatments in milk or their cognitive process in judging a product as unsafe may be less rational. Indeed, it is known that risk perception is more likely to be derived from deliberative information processing [28] and that people may have irrational and inconsistent behaviors with respect to food safety and risk information [3]. This may be stronger in highly FT neophobic respondents.

The fact that groups of respondents with different FTN did not differ in the perceived safety of items of honey could be tentatively explained as followed. Honey is generally perceived as a natural and pure product [9]. Combining this aspect with the fact that, as we already outlined, honey was the product with the lowest diversification among the three in terms of technological processing, probably the perceived naturalness was quite similar across groups and overcome the impact of technology.

## 5. Conclusions

In the current study, we showed a difference across three animal products in perceived safety. Milk was the product offering the highest commercial diversification for technologies of production (such as thermal treatment, characterizing unit operations identifying the product, etc.) and thus the one in which we observed the clearest differences in perception of safety among Italian citizens. Among milk for human consumption, items with a clear technological valence and being not highly familiar were perceived as least safe. Increasing familiarity with a product (especially characterized by peculiar technological treatment) could be an effective approach to increase the willingness to consume products from a technological transformation. For eggs, the people’s perspective (i.e., a negative perception of safety for a specific system of breeding such as ‘Battery-cage’) may push producers to slightly shift towards systems more respectful of animal welfare. In honey, some aspects related to the technology of products (mixing or blending; heating to pasteurize it) were negatively seen by citizens (perceived as less safe). However, probably due to a generalized perception of honey as natural and safe, for this product, we did not observe any effect of FTN. The organic certification achieved the highest perceived safety, when considering (eggs, honey). Independently from the product category, declaring a local/regional origin had a positive effect on the perceived safety of all products. Identified main hazards in each product category showed good awareness among citizens, in agreement with European trends. FTN clearly affected the perceived safety in items with a clear technological valence in the expected direction (that is highly FT neophobic respondents perceive ‘highly technological items’ as significantly less safe). Instead, FTN seemed not to play a role in very familiar products (for example ‘Pasteurized milk’) or in products with a high naturalness valence (honey). The results of the current study helped in better understanding how Italian citizens perceived the safety of specific items in three different product categories (milk, eggs, honey) and in clarifying the role of FTN. These results are important both for policy-making and food companies in rethinking the communication strategy concerning food safety. Specifically, stronger emphasis should be placed on the meaning of safety, and the actual contribution of technological treatment in acquiring a higher level of reliability. Moreover, the research highlights the need for new information campaigns directed to the citizens to raise awareness concerning the possible safety risks linked with a commodity such as honey, which still mantled with an aura of genuineness that overshadows possible risks and shortcomings.

## Figures and Tables

**Table 1:** Product categories and commercial types considered to evaluate the participants’ perceived safety.

Product Category	Original Italian Item	English Translation
Milk	*Latte crudo* *Latte fresco pastorizzato* *Latte microfiltrato* *Latte a lunga conservazione UHT* *Latte in polvere* *Alta-qualità*	Raw milk Pasteurized milk Microfiltered milk Ultra-High Temperature (UHT) Powder milk High-quality milk
Eggs	*Uova fresche confezionate* *Uova fresche sfuse* *Uova confezionate ‘extra-fresche’* *Uova da allevamento all’aperto* *Uova da allevamento al terra* *Uova da allevamento in gabbie* *Uova biologiche*	Fresh packaged eggs Fresh unpackaged eggs Extra fresh packaged eggs Free-range housing Cage-free housing Battery cage Organic eggs
Honey	*Miele* *Miscela di mieli* *Miele non scaldato (crudo)* *Miele in favo/con pezzi di favo* *Miele biologico*	Honey Product from mix of honeys Not heated/raw honey Honey comb Organic honey

**Table 2:** Characteristics of the respondents.

Variable	Category	Frequency (n.)	Rel. Frequency (%)
Sex	Males	163	33
	Females	322	66
Age classes (years old)	18–30	176	36
	31–50	153	31
	>50	160	33
Context	Village/rural context (<10.000 inhabitants)	154	31
	Town (10.000–70.000 inhabitants)	188	38
	City (>70.000 inhabitants)	147	30
Region *	North-West	399	82
	North-East	32	7
	Center	28	6
	South	23	5
	Islands	7	1
Diet	Flexitarian	56	13
	Omnivorous	372	83
	Vegan	3	1
	Vegetarian	16	4
Frequency of milk consumption	Daily	205	42
	Minimum 1 per week	107	22
	Minimum 1 per month	50	10
	Less than 1 per month	126	26
Frequency of eggs consumption	Daily	33	7
	Minimum 1 per week	353	73
	Minimum 1 per month	86	18
	Less than 1 per month	14	3
Frequency of honey consumption	Daily	87	18
	Minimum 1 per week	99	20
	Minimum 1 per month	120	25
	Less than 1 per month	179	37

Note: * Classification according to Nomenclature of Territorial Units for Statistics (NUTS).

**Table 3:** Effect of the food technology neophobia on the perceived safety of different products.

		Food Technology Neophobia Level							
Product Category	Commercial Type	Low		Medium		High		F	*p*-Value
Milk	Raw milk	5.0	^b^	5.5	^b^	5.9	^a^	4.77	0.009
	Pasteurized milk	7.7	^ns^	7.3	^ns^	7.3	^ns^	2.86	0.058
	UHT milk	7.8	^a^	6.9	^a^	6.5	^b^	13.85	<0.001
	Powder milk	6.6	^a^	5.6	^b^	4.7	^c^	19.71	<0.001
	Microfiltered milk	7.2	^a^	6.7	^ab^	6.4	^b^	4.64	0.010
	High-quality milk	7.6	^ns^	7.3	^ns^	7.2	^ns^	2.40	0.092
Eggs	Fresh packaged eggs	6.8	^a^	6.1	^b^	6.1	^b^	7.66	<0.001
	Extra fresh packaged eggs	7.1	^a^	6.3	^b^	6.3	^b^	8.42	<0.001
	Fresh unpackaged eggs	6.2	^ns^	5.8	^ns^	5.7	^ns^	2.1	0.124
	Organic eggs	7.4	^ns^	7.1	^ns^	7.2	^ns^	1.88	0.154
	Free-range housing	7.1	^a^	6.7	^b^	6.8	^ab^	2.88	0.057
	Cage-free housing	6.7	^ns^	6.4	^ns^	6.3	^ns^	2.57	0.077
	Battery cage	4.7	^a^	4.3	^ab^	3.7	^b^	5.49	0.004
Honey	Honey	7.4	^ns^	7.2	^ns^	7.4	^ns^	1.06	0.349
	Products from a mix of honeys	5.8	^ns^	5.5	^ns^	5.2	^ns^	1.89	0.152
	Organic honey	7.6	^ns^	7.5	^ns^	7.8	^ns^	1.56	0.211
	Not-heated/raw honey	6.8	^ns^	6.6	^ns^	7.0	^ns^	1.92	0.148
	Honeycomb	6.5	^ns^	6.3	^ns^	6.7	^ns^	1.50	0.223

Within each row, different letters indicate significant different mean values from one-way ANOVA models separately conducted to assess the effect of FTN (low, medium, high) on the perceived safety of each product category (fixed factor: level of technological transformation or commercial type).

## Data Availability

Data is contained within the article and supplementary material.

## Supplementary Materials

The following supporting information can be downloaded at: https://www.mdpi.com/article/10.3390/foods11223739/s1, Table S1: Complete ANOVA models and effect of size (eta^2^ = SS factor/ SS total; partial eta^2^ = SS model/ (SS model + SS error)), including fixed models (a) and mixed models (b).

## Abbreviations

FTN = Food Technology Neophobia; FTNS = Food technology Neophobia Scale; FT = Food Technology; CATA = Check-All-That-Apply test; UHT-milk = Ultra High-Temperature treated milk; EU = European Union.
