# Supplementary material for: Understanding Italian Consumers’ Perception of Safety in Animal Food Products
Source: Foods. 2022 Nov 21;11(22):3739. doi: 10.3390/foods11223739 (PMC9689367; doi:10.3390/foods11223739)
Supplement: Supplementary file 1 [file foods-11-03739-s001.zip › foods-1991451-supplementary.pdf]

foods MDPI

Supplementary material

Table S1. Complete ANOVA models and effect of size (eta^2 = SS factor/ SS total; partial eta^2 = SS model/ (SS model + SS error)), including fixed models (a) and mixed models (b).

a). Fixed ANOVA models (output of Type III Sum of Squares for variable 'perceived safety'):

	Source	DF	Sum of squares (SS)	Mean of squares	F	p value	eta^2	Partial eta^2
Sex	product	2.00	389.53	194.76	48.40	0.000	0.59	0.02
	sex	1.00	247.89	247.89	61.60	0.000	0.38	
	product*sex	2.00	21.17	10.59	2.63	0.072	0.03	
		tot:	658.59					
Age	product	2.00	461.04	230.52	57.01	0.000	0.58	0.02
	age_classes	2.00	326.68	163.34	40.40	0.000	0.41	
	product*age_classes	4.00	8.66	2.17	0.54	0.710	0.01	
		tot:	796.38					
Context	product	2.00	469.22	234.61	57.57	0.000	0.89	0.01
	context	2.00	16.30	8.15	2.00	0.135	0.03	
	product*context	4.00	41.48	10.37	2.54	0.038	0.08	
		tot:	527.01					
Diet	product	2.000	151.399	75.700	18.755	<0,001	0.18	0.02
	diet	3.000	408.668	136.223	33.750	<0,001	0.48	
	product*diet	6.000	298.057	49.676	12.308	<0,001	0.35	
		tot:	858.124					

b). Mixed Models (output of Type III Sum of Squares for variable 'perceived safety'):

	Source	Type	DF	Sum of squares (SS)	Mean squares	E(Mean squares)	F	p value	eta^2	Partial eta^2
Milk type	subject	Random	488.00	5454.33	11.18	sigma2 + 35.6356 * sigma2(subject)	5.10	<0,001	0.44	0.55
	Milk type	Fixed	5.00	1719.54	343.91	sigma2 + 2904.3 * Q(type)	156.81	<0,001	0.14	

	Error		2440.00	5351.29	2.19	sigma2			0.43	
			tot:	12525.17						
Egg type	subject	Random	464.00	6398.19	13.79	sigma2 + 50.8726 * sigma2(subject)	7.92	<0,001	0.45	0.64
	Egg type	Fixed	6.00	2572.23	428.71	sigma2 + 3553.82 * Q(type)	246.27	<0,001	0.18	
	Error		2952.00	5138.77	1.74	sigma2			0.36	
			tot:	14109.20						
Honey type	subject	Random	464.00	4391.80	9.47	sigma2 + 26.0661 * sigma2(subject)	5.29	<0,001	0.47	0.62
	Honey type	Fixed	4.00	1331.58	332.90	sigma2 + 2549.26 * Q(type)	186.07	<0,001	0.14	
	Error		1976.00	3535.27	1.79	sigma2			0.38	
			tot:	9258.65						
Milk origin	subject	Random	464.00	3484.56	7.51	sigma2 + 16.7179 * sigma2(subject)	3.96	<0,001	0.33	0.73
	origin	Fixed	3.00	4121.65	1373.88	sigma2 + 2043.77 * Q(origin)	724.21	<0,001	0.40	
	Error		1488.00	2822.85	1.90	sigma2			0.27	
			tot:	10429.06						
Eggs origin	subject	Random	464.00	3398.87	7.33	sigma2 + 16.7179 * sigma2(subject)	4.20	<0,001	0.34	0.74
	origin	Fixed	3.00	3994.16	1331.39	sigma2 + 2043.77 * Q(origin)	763.11	<0,001	0.40	
	Error		1488.00	2596.09	1.74	sigma2			0.26	
			tot:	9989.12						
Honey origin	subject	Random	464.00	3719.10	8.02	sigma2 + 16.7179 * sigma2(subject)	4.54	<0,001	0.37	0.74
	origin	Fixed	3.00	3682.75	1227.58	sigma2 + 2043.77 * Q(origin)	695.85	<0,001	0.37	
	Error		1488.00	2625.06	1.76	sigma2			0.26	
			tot:	10026.91						
